# Socio-economic impacts of brucellosis on livestock production and reproduction performance in Koibatek and Marigat regions, Baringo County, Kenya

**DOI:** 10.1186/s12917-020-02283-w

**Published:** 2020-02-18

**Authors:** Peter N. Lokamar, Moses A. Kutwah, Harrysone Atieli, Sussy Gumo, Collins Ouma

**Affiliations:** 1 Department of Public Health, School of Public Health and Community Development, Maseno University, Kisumu, Kenya; 2 Department of Disease Surveillance and Epidemic Response, Ministry of Health, Nairobi, Kenya; 3 Kenya Nutritionists and Dieteticians Institute, Nairobi, Kenya; 4 Department of Religion, Theology and Philosophy, School of Arts and Social Sciences, Maseno University, Kisumu, Kenya; 5 Department of Biomedical Sciences and Technology, School of Public Health and Community Development, Maseno University, Kisumu, Kenya

**Keywords:** Brucellosis, Farmers, Perceived impact, Baringo County, Kenya

## Abstract

**Background:**

Brucellosis in Africa is caused by Brucella species transmitted through contaminated or contacts with infected animals or their carcasses. The disease reduces livestock production and reproduction performance evident by frequent episodes of abortion, still births, swollen testes, weak calves/lambs and swollen joints. However, the socio-economic impacts of these brucellosis-associated symptoms on milk, fat, meat and blood production, infertility, sale value, dowry and costs of treatment has not been evaluated extensively in developing countries. In Baringo County, Kenya, there is a continuous movement of cattle as a result of trade and grazing, which predisposes many herds to brucellosis infection. The objective of this study was to investigate the socio-economic impacts of Brucella infection on production systems for sheep, goats, cattle and camels and explore the impact of brucellosis on livestock production and reproduction performance among livestock keeping communities in Baringo County, Kenya. The study adopted a cross-sectional survey using quantitative data collection methods.

**Results:**

Results demonstrated an impact on milk production in suspected brucellosis cases resulting from abortions (OR = 0.151, *P* < 0.0001) and swollen joints (OR = 2.881, *P* < 0.0001). In terms of infertility, abortion as a symptom of brucellosis (OR = 0.440, *P* = 0.002), still birth (OR = 0.628, *P* = 0.042), and weak calf or lamb (OR = 0.525, *P* = 0.005) had an impact on infertility. In terms of sale value, abortion (OR = 0.385, *P* = 0.008), weak calf/lamb (OR = 2.963, *P* = 0.013) had an impact on sale value. Other analyses demonstrated that for dowry, swollen testes (OR = 5.351, *P* = 0.032), weak calf and lambs (OR = 0.364, *P* = 0.019) had a likelihood of reduction of dowry value. Finally, in terms of cost of treatment, abortion (OR = 0.449, *P* = 0.001), still births (OR = 0.208, *P* = 0.015), swollen testes (OR = 0.78, *P* = 0.014), weak calf/lambs (OR = 0.178, *P* = 0.007) and swollen joints (OR = 0.217, *P* = 0.003) significantly increased the costs of treatments. There was no impact on fat and meat and blood production.

**Conclusion:**

Even though there was a huge socio-economic impact on milk production, infertility, sale value, and dowry, it was the costs of treatment that was significantly impacted on all symptoms associated with brucellosis on this community. A ‘One Health’ approach in tackling the brucellosis menace as a holistic approach is recommended for both humans and their livestock.

## Introduction

Globally, brucellosis is described as a highly contagious zoonotic disease, and a cause of significant reproductive losses in livestock [1]. In Africa, brucellosis is described as enzootic and is common in low- and middle-income countries (LMICs). The disease caused by Brucella species (*B. abortus, B. mellitensis,* and *B. suis*) is transmitted through contaminated and unpasteurized milk, milk products or by direct contact with infected animals or animal carcasses [2]. Abortion constituents, uterine exudates and colostrum are highly infectious [3]. Animal brucellosis causes direct socio-economic effects in communities who depend on animal production for their livelihood. Losses in animals are attributed to direct effects on their offspring due to abortion, stillbirth and infertility whereas indirect losses are due to reduction in milk yields and humans suffering resulting from the disease [4].

In LMICs, the prevalence of animal and human brucellosis is generally unknown due to a myriad of challenges with diagnostics, reporting and weak to non-existent surveillance systems, especially in malaria endemic areas [5, 6] with variations based on the pastoral systems.

Although prevalence is high and variable in many countries, surveillance for the disease is generally poor [7]. Factors assumed to be responsible for variation in prevalence include purchase of infected cattle from the market for replacement or upgrading, nature of animal production, sharing of bulls, use of open-range grazing, demographic factors, regulatory issues, and climate and wildlife interaction [7]. Furthermore, one major factor contributing to the spread of the disease is the free movement of animals practiced by the livestock keepers in regions such as in Baringo County, Kenya. Despite under-reporting and inadequate epidemiologically valid data, the evidence obtained throughout the years illustrate that brucellosis is a widespread problem in Africa, a continent where several Sub-Saharan countries are estimated to bear a high burden of neglected zoonotic diseases [2, 8].

In terms of socio-economic effects, it has been documented that most quantifiable expressions of Brucella are linked to reproduction [2]. For example, infected male animals were prone to infertility and reduced reproductive performance. Female animals, on the other hand, suffered from abortion, stillbirth and early death of offspring when the uterus gets infected. In addition to spreading the infection to humans, animal brucellosis impacts livestock productivity, which can have adverse socio-economic and indirect health consequences on humans, especially helpless livestock-keeping populations in resource-limited surroundings that depend on livestock for food security and income [2].

The impacts of brucellosis in livestock include abortion and death as well as decreased milk and meat production and reduced reproductive efficiency [9]. Generally, the costs associated with the treatment in animals attributed to diseases such as brucellosis is remarkably high [10, 11]. As the disease is hardly remarkable in its chronic stage and despite the losses and yield decrease, its causes often goes unnoticed. Its negative effect on cost-effectiveness of livestock production is extremely undervalued particularly in tropical areas in wide-ranging management system [12]. Brucellosis illness to the herds reduces livestock production and reproduction performance evident by frequent episodes of abortion especially during the last trimester, retention of placenta, metritis, birth of weak calves, infertility in bulls and cows and 20% reduction in milk production from infected cows [13, 14].

In Baringo County, where the current study was conducted, brucellosis prevalence is unknown due to lack of awareness among communities about the disease, but more importantly due to weakened animal and public health systems owing to the remoteness of the region and insecurity. As a consequence, the disease remains largely neglected with little attention given to prevention and control in livestock and humans [15]. In this region of study, there is a continuous movement of cattle as a result of trade and for grazing, which then predisposes many herds to brucellosis infection [15]. Owing to the difficulty in gathering accurate data on the persistence and disease prevalence of brucellosis in pastoral communities the information remains scarce. Studies on the socio-economic effects of Brucella infection on reproductive conditions are generally rare in Kenya. As much as it is widely known that infection with brucellosis has socio-economic impacts, the actual impact on livestock production and reproduction performance in agrarian and nomadic regions in Baringo County remain unestablished. As such the objective of this study was to investigate the socio-economic impacts of Brucella infection on production systems of indigenous, mixed and exotic breeds (sheep, goats, cattle and camels) and explore the impact of brucellosis on livestock production and reproduction performance among livestock keeping communities in Baringo County, Kenya.

## Results

### Response rate

The final sample size obtained was 640 herds; (320 bovine, 106 camel, 154 goat and 60 sheep) from 604 households.

### Demographic characteristics of the study population

This study targeted households with domestic ruminants; cattle, goats, sheep and camels. Livestock keepers who were in close contact with the animals were the key respondents for interview and were enrolled as study participants. The study was conducted in Baringo County and targeted 8 locations in Koibatek and Marigat sub-counties; Torongo, Koibatek, Ravine, Lembus Kwen, Marigat, Eldume, Kimalel and Loboi. The study revealed that 67.5% of residents in Marigat Sub-county and 32.5% of residents in Koibatek Sub-county reared domestic ruminants. Majority of the animals reared were females 54.5% (*n* = 349). Most of the animals reared were aged ≥3 years, 43.0% (*n* = 275), 2–3 years 38.1% (*n* = 244) and a few aged 2 years and below 18.9% (*n* = 121). Domesticated animals were as follows: 68.3% were indigenous breeds, 74.3% utilized natural breeding system and 34.4% adopted mixed farming as the primary type of production system. The main source of drinking water for livestock was river (37.7%) and borehole (36.6%) (Table 1).

**Table 1:** Demographic Characteristics of the Study Population

Characteristic *(n = 640)*	Frequency	Percentage (%)
Species per Sub-county		
**Marigat**	**432**	**67.5%**
Bovine	114	26.4%
Sheep	58	13.4%
Goat	154	35.6%
Camel	106	24.5%
**Koibatek**	**208**	**32.5%**
Bovine	206	99.0%
Sheep	2	1.0%
Goat	0	0%
Camel	0	0%
Gender of the animals		
Male	291	45.5%
Female	349	54.5%
Age of the species in years		
**Bovine**	**320**	**50.0%**
< 2 years	47	38.8%
2–3 years	109	44.7%
> 3 years	164	59.6%
**Sheep**	**60**	**9.4%**
< 2 years	13	10.7%
2–3 years	29	11.9%
> 3 years	18	6.5%
**Goat**	**154**	**24.1%**
< 2 years	45	37.2%
2–3 years	66	27.0%
> 3 years	43	15.6%
**Camel**	**106**	**16.6%**
< 2 years	16	13.2%
2–3 years	40	16.4%
> 3 years	50	18.2%
Types of breeds	(Cumulative frequencies)	
Indigenous	441	68.3%
Exotic	205	31.7%
Type of production system		
Nomadic pastoralist	218	23.6%
Agro-pastoralist	135	14.6%
Mixed farming	318	34.4%
Commercial ranch	8	0.9%
Peri-urban	25	2.7%
Semi-zero grazing	220	23.8%
Type of breeding system		
Artificial insemination	176	25.7%
Natural	508	74.3%
Main source of drinking water for livestock		
Pond	63	9.8%
Borehole	234	36.6%
River	241	37.7%
Compound trough	102	15.9%

Presented are N (%) for all categories

### Reported socio-economic impact of brucellosis on livestock production and reproduction performance

The association between socio-economic factors and abortion, still birth, retained placenta, swollen joints, swollen testes, weak calf or lamb, repeat breeder and apparent infertility as consequences of brucellosis was performed. In addition, the perceived socio-economic impact on milk, meat and blood, fat, infertility, sale value, dowry and cost of treatment against abortion was assessed. Results revealed that the proportions of those who indicated that brucellosis had an impact on milk production was either medium (54.1%) or high (6.8%), meat and blood as medium (54.4%) or high (5.7%), infertility (66.0%), sale value as medium (64.3%) or high (5.7%), dowry (50.1%) and cost of treatment as medium (62.3%) or high (33.7%). All these proportions were significantly higher (all at *P* < 0.0001). However, the proportions of those who said the effects was high, medium or had no impact on fat production were comparable (*P* = 0.127).

Further regression analyses indicated that there was 85% impact on milk production in suspected brucellosis cases (OR = 0.151, 95% CI = 0.066–0.342, *P* < 0.0001). Furthermore, results demonstrated that there was 56% impact on infertility (OR = 0.440, 95% CI = 0.261–0.743, *P* = 0.002), 62% reduction on sale value (OR = 0.385, 95% CI = 0.190–0.776, *P* = 0.008), 56% increment on cost of treatment (OR = 0.449, 95% CI = 0.281–0.717, *P* = 0.001). Even though it indicated that there was significantly no loss to fat (OR = 3.156, 95% CI = 1.152–8.646, *P* = 0.025), there was no effect on dowry (*P* = 0.829) and meat and blood production (*P* = 0.220) (Table 2).

**Table 2:** Reported impact of abortion on socio-economic status

Variables	Abortions^a^				*χ*^*2*^	*P-value*	*OR*	*95% CI*	*P-value*
	Yes		No						
	*n*	*%*	*n*	*%*					
Milk									
High	24	6.8	86	30.0	62.9	< 0.0001*	0.151	0.066–0.342	< 0.0001*
Medium	191	54.1	133	46.3					
Low	138	39.1	68	23.7					
Meat and blood									
High	20	5.7	58	20.2	36.4	< 0.0001*	0.541	0.203–1.444	0.220
Medium	192	54.4	154	53.7					
Low	141	39.9	75	26.1					
Fat									
High	15	4.2	21	7.3	4.1	0.127	3.156	1.152–8.646	0.025*
Medium	185	52.4	158	55.1					
Low	153	43.3	108	37.6					
Infertility									
High	100	28.3	134	46.7	23.1	< 0.0001*	0.440	0.261–0.743	0.002*
Medium	133	37.7	77	26.8					
Low	120	34.0	76	26.5					
Sale value									
High	20	5.7	67	13.6	48.7	< 0.0001*	0.385	0.190–0.716	0.008*
Medium	227	64.3	126	55.2					
Low	106	30.0	94	31.3					
Dowry									
High	16	4.5	37	12.9	19.6	< 0.0001*	0.912	0.396–2.101	0.829
Medium	161	45.6	143	49.8					
Low	176	49.9	107	37.3					
Cost of treatment									
High	119	33.7	159	55.4	34.5	< 0.0001*	0.449	0.281–0.717	0.001*
Medium	220	62.3	112	39.0					
Low	14	4.0	16	5.6					

^a^Pearson Chi-square. *Statistically significant at *P* ≤ 0.05. *OR* odds ratio, *95% CI* 95% Confidence Interval. OR generated through logistic regression analyses

Perceived socio-economic impact associated with still birth as a result of brucellosis on milk, meat and blood, infertility, sale value, dowry and cost of treatment was assessed. Results showed the proportions of those who indicated that stillbirth had an impact on the following; milk as medium (64.9%) or high (6.9%), meat and blood, medium (64.9%) or high (4.8%), sale value as medium (71.4%) or high (5.6%), cost of treatment as medium (67.5%) or high (31.2%), infertility (66.7%) and dowry (58.9%). All these proportions were significant (all *P* < 0.0001). However, the proportion of respondents who said still birth had high, medium or had no impact on fat (60.6%) was comparable at (*P* = 0.062) (Table 3).

**Table 3:** Reported impact of still birth on socio-economic status

Variables	Still birth^a^				*χ*^*2*^	*P-value*	*OR*	*95% CI*	*P-value*
	Yes		No						
	*n*	*%*	*n*	*%*					
Milk									
High	16	6.9	94	23.0	38.6	< 0.0001*	2.122	0.913–4.933	0.800
Medium	150	64.9	174	42.5					
Low	65	28.1	141	34.5					
Meat and blood									
High	11	4.8	67	16.4	25.5	< 0.0001*	0.854	0.501–1.455	0.560
Medium	150	64.9	196	47.9					
Low	70	30.3	146	35.7					
Fat									
High	7	3.0	29	7.1	5.6	0.062*	1.139	0.706–1.836	0.594
Medium	133	57.6	210	51.3					
Low	91	39.4	170	41.6					
Infertility									
High	49	21.2	185	45.2	41.7	< 0.0001*	0.628	0.402–0.983	0.042*
Medium	105	45.5	105	25.7					
Low	77	33.3	119	29.1					
Sale value									
High	13	5.6	74	18.1	42.2	< 0.0001*	0.684	0.416–1.123	0.133
Medium	165	71.4	188	46.0					
Low	53	22.9	147	35.9					
Dowry									
High	8	3.5	45	11.0	15.7	< 0.0001*	1.091	0.674–1.767	0.723
Medium	128	55.4	176	43.0					
Low	95	41.1	188	46.0					
Cost of treatment									
High	72	31.2	206	50.4	38.5	< 0.0001*	0.208	0.059–0.738	0.015*
Medium	156	67.5	176	43.0					
Low	3	1.3	27	6.6					

^a^Pearson Chi-square. *Statistically significant at *P* ≤ 0.05. *OR* odds ratio, *95% CI* 95% Confidence Interval. OR generated through logistic regression analyses

Logistics regression model with still birth as dependent variable was established to determine socio-economic status influence on still birth. There was 38% impact on infertility (OR = 0.628, 95% CI = 0.402–0.983, *P* = 0.042) and 80% increment on cost of treatment in suspected brucellosis cases (OR = 0.208, (95% CI = 0.059–0.738, *P* = 0.015). On the contrary, no significant effect was attributed to milk production (*P* = 0.800), meat and blood production (*P* = 0.560), fat production (*P* = 0.594), dowry (*P* = 0.723) and sale value (*P* = 0.133) (Table 3).

We further assessed the socio-economic impact of swollen testes as a sign of suspected brucellosis on the same products: milk, meat and blood, infertility, sale value, dowry and cost of treatment. The following were the proportions of those who specified that swollen testes had impact on production: milk (67.3%), infertility (64.2%), dowry (56.6%), meat and blood as medium (63.5%) or high (5.0%), fat as medium (61.7%) or high (2.9%), sale value as medium (71.1%) or high (5.7%), cost of treatment as medium (66.7%) or high (32.7%). The proportions of those who indicated that it had an impact on sale value (*P* < 0.0001), dowry (*P* < 0.0001) and cost of treatment (*P* < 0.0001), meat and blood (*P* = 0.002), milk (*P* = 0.004) and infertility (*P* = 0.003) was significantly higher than those who indicated it had no impact. However, the proportions of those who said fat (61.7%) had or had no impact on swollen testes was comparable (*P* = 0.100) (Table 4).

**Table 4:** Reported impact of swollen testes on socio-economic status

Variables	Swollen testes^a^				*χ*^*2*^	*P-value*	*OR*	*95% CI*	*P-value*
	Yes		No						
	*n*	*%*	*n*	*%*					
Milk									
High	14	8.8	96	20.0	11.3	0.004*	1.688	0.964–2.957	0.670
Medium	93	58.5	231	48.0					
Low	52	32.7	154	32.0					
Meat and blood									
High	8	5.0	70	14.6	12.7	0.002*	0.942	0.525–1.689	0.841
Medium	101	63.5	245	50.9					
Low	50	31.4	166	34.5					
Fat									
High	2	1.3	34	7.1	9.3	0.100	0.730	0.435–1.225	0.233
Medium	96	60.4	247	51.4					
Low	61	38.4	200	41.6					
Infertility									
High	40	25.2	194	40.3	11.9	0.003*			
Medium	62	39.0	148	30.8			1.046	0.644–1.696	0.857
Low	57	35.8	139	28.9					
Sale value									
High	9	5.7	78	16.2	23.8	< 0.0001*	0.641	0.365–1.125	0.122
Medium	113	71.1	240	49.9					
Low	37	23.3	163	33.9					
Dowry									
High	2	1.3	51	10.6	15.4	< 0.0001*	5.351	1.159–24.706	0.032*
Medium	88	55.3	216	44.9					
Low	69	43.4	214	44.5					
Cost of treatment									
High	52	32.7	226	47.0	21.9	< 0.0001*	0.078	0.010–0.598	0.014*
Medium	106	66.7	226	47.0					
Low	1	0.6	29	6.0					

^a^Pearson Chi-square. *Statistically significant at *P* ≤ 0.05. *OR* odds ratio, *95% CI* 95% Confidence Interval. OR generated through logistic regression analyses

Logistics regression analysis established that there was 5 times more impact on dowry (OR = 5.351, 95% CI = 1.159–24.706, *P* = 0.032) and 22% increment on cost of treatment (OR = 0.78, 95% CI = 0.10–0.598, *P* = 0.014). Even so, the proportions of those who indicated that it had impact versus no impact were comparable between loss in milk production (*P* = 0.670), meat and blood production (*P* = 0.841), fat production (*P* = 0.233), infertility (*P* = 0.857) and sale value (*P* = 0.122) in animals suspected to suffer from brucellosis (Table 4).

We additionally reported on the socio-economic impact related to weak calf or lamb as a result of suspected brucellosis on milk, meat and blood, infertility, sale value, dowry and cost of treatment. Results exhibited that the proportions of those who specified that weak calf or lamb had impact on were significantly higher for milk as medium (61.7%) or high (8.7%) meat and blood as medium (63.5%) or high (5.6%), sale value as medium (66.5%) or high (5.2%), cost of treatment as medium (63.9%) or high (34.8%), infertility (73.5%), and fat (60.2%) (all *P* < 0.0001), while for fat it was at *P* = 0.029. However, the proportions of those who said dowry (61.3%) had or had no impact on weak calf or lamb was comparable (*P* = 0.052) (Table 5).

**Table 5:** Reported impact of weak calf or lamb on socio-economic status

Variables	Weak calf or lamb^a^				*χ*^*2*^	*P-value*	*OR*	*95% CI*	*P-value*
	Yes		No						
	*n*	*%*	*n*	*%*					
Milk									
High	20	8.7	90	22.0	24.6	< 0.0001*	1.113	0.668–1.855	0.680
Medium	142	61.7	182	44.3					
Low	68	29.6	138	33.7					
Meat and blood									
High	13	5.6	65	15.9	19.4	< 0.0001*	0.818	0.477–1.401	0.464
Medium	146	63.5	200	48.8					
Low	71	30.9	145	35.3					
Fat									
High	7	3.0	29	7.1	7.1	0.029*	1.462	0.501–4.261	0.487
Medium	136	59.2	207	50.5					
Low	87	37.8	174	42.4					
Infertility									
High	70	30.5	164	40.0	17.1	< 0.0001*	0.525	0.333–0.827	0.005*
Medium	99	43.0	111	27.1					
Low	61	26.5	135	32.9					
Sale value									
High	12	5.2	75	18.3	27.9	< 0.0001*	2.963	1.258–6.982	0.013*
Medium	153	66.5	200	48.8					
Low	65	28.3	135	32.9					
Dowry									
High	17	7.4	36	8.8	6.0	0.052	0.364	0.157–0.845	0.019*
Medium	124	53.9	180	43.9					
Low	89	38.7	194	47.3					
Cost of treatment									
High	80	34.8	198	48.3	24.9	< 0.0001*	0.178	0.051–0.622	0.007*
Medium	147	63.9	185	45.1					
Low	3	1.3	27	6.6					

^a^Pearson Chi-square. *Statistically significant at *P* ≤ 0.05. *OR* odds ratio, *95% CI* 95% Confidence Interval. OR generated through logistic regression analyses

This assessment further explored on how weak calf/lamb influenced socio-economic status of the households. There was 64% likelihood of reduction of dowry value (OR = 0.364, 95% CI = 0.157–0.845, *P* = 0.019), 2 times reduction in sale value (OR = 2.963, 95% CI = 1.258–6.982, *P* = 0.013), 48% increased chances of infertility (OR = 0.525, 95% CI = 0.333–0.827, *P* = 0.005) and 83% possibility on increment in cost of treatment (OR = 0.178, 95% CI = 0.051–0.622, *P* = 0.007) in suspected brucellosis instances. From the model, there was no significant effect on milk production (*P* = 0.680), meat and blood production (*P* = 0.464) and fat production (*P* = 0.487) (Table 5).

We finally assessed the socio-economic impact on milk, meat and blood, fat, infertility, sale value, dowry and cost of treatment as a result of swollen joint, as part of suspected brucellosis. The outcome demonstrated that the proportions of those who pointed out that brucellosis had an impact of milk production was (43.1%), meat and blood (49.0%), sale value (53.9%) and dowry (46.1%). Infertility was reported as medium (32.3%) and high (52.0%) whereas the proportion of those indicating cost of treatment being medium (39.2%) and high (59.8%). All these proportions were significantly higher (*P* < 0.0001) relative to those who said there was no impact. However, the proportions of those who said brucellosis had or had no impact on fat production were comparable (*P* = 0.114) (Table 6).

**Table 6:** Reported impact of swollen joints on socio-economic status

Variables	Swollen joints^a^				*χ*^*2*^	*P-value*	*OR*	*95% CI*	*P-value*
	Yes		No						
	*n*	*%*	*n*	*%*					
Milk									
High	13	12.7	24	12.8	28.4	< 0.0001*	2.881	1.733–4.790	< 0.0001*
Medium	31	30.4	114	6.06					
Low	58	56.9	50	26.6					
Meat and blood									
High	12	11.8	22	11.7	16.1	< 0.0001*	1.561	0.921–2.644	0.980
Medium	38	37.2	113	60.1					
Low	52	51.0	53	28.2					
Fat									
High	7	6.9	11	5.9	4.3	0.114	0.679	0.432–1.067	0.093
Medium	49	48.0	114	60.6					
Low	46	45.1	63	33.5					
Infertility									
High	53	52.0	48	25.5	22.1	< 0.0001*	1.126	0.720–1.762	0.603
Medium	33	32.3	78	41.5					
Low	16	15.7	62	33.0					
Sale value									
High	11	10.8	19	10.1	19.8	< 0.0001*	0.758	0.464–1.236	0.267
Medium	44	43.1	128	68.1					
Low	47	46.1	41	21.8					
Dowry									
High	12	11.8	14	7.4	13.5	0.001*	1.093	0.684–1.746	0.711
Medium	35	34.3	107	56.9					
Low	55	53.9	67	25.6					
Cost of treatment									
High	61	59.8	59	31.4	22.5	< 0.0001*	0.217	0.079–0.594	0.003*
Medium	40	39.2	122	64.9					
Low	1	1.0	7	3.7					

^a^Pearson Chi-square. *Statistically significant at *P* ≤ 0.05. *OR* odds ratio, *95% CI* 95% Confidence Interval. OR generated through logistic regression analyses

Further logistic regression analyses demonstrated almost 3 times likelihood of reduction in milk production (OR = 2.881, 95% CI = 1.733–4.790, *P* < 0.0001) and a 79% likely increase in cost of treatment (OR = 0.217, 95% CI = 0.79–0.594, *P* = 0.003) in animals suspected to be ailing from brucellosis. However, there was no association on meat and blood (*P* = 0.980), fat (*P* = 0.093), infertility (*P* = 0.603), sale value (*P* = 0.267) and dowry (*P* = 0.711) (Table 6).

## Discussion

Brucellosis is considered a neglected disease that significantly affects countries where resources are limited, and as such, there are only a few studies that has measured the socio-economic impact of brucellosis in livestock. Even though the estimated socio-economic impacts vary with the location, production system, facilities, and miscellaneous factors including indirect health effects of the disease in humans [16], the current study focused on respondent’s perceived socio-economic impact of brucellosis on livestock production and reproduction performance with a focus on milk, meat and blood, and fat production, infertility, sale value, dowry and cost of treatment relative to suspected brucellosis symptoms (abortions, still births, swollen testes, weak calf or lamb, and swollen joints). The current study was focused in Baringo County, since in this region, brucellosis prevalence is unknown due to weakened animal and public health systems majorly attributed by the remoteness of the region and a huge insecurity making it almost impossible to carry out detailed analyses on the Brucella disease dynamics in the population. Consequently, the disease still remains essentially neglected with little attention given to prevention and control in livestock and humans, with a continuous movement of cattle, which then predisposes many herds to brucellosis infection [15].

Our results demonstrated that there was 85% impact on milk production in suspected brucellosis cases resulting from abortions (OR = 0.151, 95% CI = 0.066–0.342, *P* < 0.0001) and almost 3 times likelihood of reduction in milk production (OR = 2.881, 95% CI = 1.733–4.790, *P* < 0.0001) as a result of swollen joints. However, there was no impact of stillbirth, swollen testes and weak calf and lambs on milk production as reported by farmers in this region. In previous studies carried out in other settings [17], it was demonstrated that mastitis suspected to be resulting from brucellosis infection led to reduction in milk production in cattle. Even though mastitis was not evaluated in the current study, the findings in the previous study is generally consistent with our observations that suspected brucellosis infection symptoms are directly related to reduced mild production. Other observations in Tanzania have noted that despite immunizations with *Brucella* (*B*.) *abortus* RB51 vaccine, a rise in abortions suspiciously caused by *Brucella* was still eminent in a dairy cattle herd [18]. They further indicated that the disease has serious economic implications resulting from abortions, infertility and decreased milk production, thus necessitating the implementation of surveillance and control strategies to forestall the socio-economic effects in both developed and developing countries where the disease is endemic.

In terms of infertility, our study demonstrated that abortion as a symptom of brucellosis had a 56% impact on infertility (OR = 0.440, 95% CI = 0.261–0.743, *P* = 0.002), there was a 38% impact on infertility (OR = 0.628, 95% CI = 0.402–0.983, *P* = 0.042) resulting from still birth, and 48% increased chances of infertility (OR = 0.525, 95% CI = 0.333–0.827, *P* = 0.005) due to weak calf or lamb. Even though, swollen testes and swollen joints as symptoms of brucellosis had no impact on infertility, our findings that symptoms associated with brucellosis are consistent with earlier in vitro studies on rat models that demonstrated that *B. abortus* infection induced 41.67% infertility in the infected rats [19]. In the study conducted in Korea, they concluded that *B. abortus* biotype 1 infections in rat models affect reproduction adversely by causing infertility, stillbirth and loss of number and weight of offspring. To date, no studies has been carried out extensively on the impact of brucellosis on livestock. We report for the first time that livestock with suspected brucellosis infection have increased risk to infertility potentially resulting from abortion- and still birth-related complications.

In terms of sale value, there was 62% reduction on sale value (OR = 0.385, 95% CI = 0.190–0.776, *P* = 0.008) resulting from abortion, and 3 times reduction in sale value (OR = 2.963, 95% CI = 1.258–6.982, *P* = 0.013) resulting from weak calf/lamb. However, stillbirth, swollen testes and swollen joints had no impact on sale value in this region. Other analyses demonstrated that for dowry, which is a form of appreciation and which is usually provided in the form of livestock in this set-up, there was 5 times more impact on dowry (OR = 5.351, 95% CI = 1.159–24.706, *P* = 0.032) whenever they had swollen testes, 64% likelihood of reduction of dowry value (OR = 0.364, 95% CI = 0.157–0.845, *P* = 0.019) resulting from weak calf and lambs. However, abortion, stillbirth and swollen joints as symptoms of suspected brucellosis had no impact on dowry. Even though not evaluated previously, we have established that livestock with symptoms associated with brucellosis had a significant reduction in sale value, more so if they had documented abortion and weak calves or lambs. This is tied more closely to payment of dowry in this community as they always conduct a thorough examination on livestock intended for the purposes of dowry payment by carrying out physical examination and asking historical questions about their offspring. All these approaches, as they may seem trivial at face-value, add up to form strong and deeply engrained attitudes that are based on culture and tradition, which then affect the socio-economic status of the families involved. More studies, however, need to be conducted to further assess the impacts on sale value and dowry in the context of a variety of nomadic and non-nomadic communities, especially in areas where the true prevalence of brucellosis has been established.

Finally, in terms of cost of treatment, there was 56% increment on cost of treatment (OR = 0.449, 95% CI = 0.281–0.717, *P* = 0.001) resulting from abortion, 80% increment on cost of treatment in suspected brucellosis cases (OR = 0.208, 95% CI = 0.059–0.738, *P* = 0.015) resulting from still births, 22% increment on cost of treatment (OR = 0.78, 95% CI = 0.10–0.598, *P* = 0.014) resulting from swollen testes, 83% increment in cost of treatment (OR = 0.178, 95% CI = 0.051–0.622, *P* = 0.007) resulting from weak calf or lamb and a 79% likely increase in cost of treatment (OR = 0.217, 95% CI = 0.79–0.594, *P* = 0.003) resulting from swollen joints. This demonstrates that the cost of treatment significantly increases in the presence of all the symptoms associated with brucellosis in this community. A number of economic studies have been conducted in other countries highlighting the potential losses for livestock producers and the general economy from brucellosis, with the largest losses mainly affecting developing nations [2]. However, the costs of treatment as an impact still remains elusive in developing countries due to lack of comprehensive field and experimental data. Even though we established from the respondents that all the brucellosis symptoms tested in the current study (i.e. abortion, still births, swollen testes, weak calves/lambs and swollen joints) had a higher likelihood of having an increased cost of treatment, a further model incorporating both animal and human aspects in a longitudinal set-up need to be developed. The results are, however, consistent with other observations [20] that showed a significant impact on the costs of treatment on brucellosis-infected yaks in Tibet. Even though not assessed in the current study, respondents who mentioned high costs involved in the treatment of brucellosis were less likely to choose a government health facility compared to a private health facility [21], a pointer that there are huge costs involved in the treatment of brucellosis.

Even though some of the symptoms of suspected brucellosis were not associated with any of the production (e.g. fat, meat and blood production) and reproduction performance, we fully agree with the previous study that highlights a ‘One Health’ approach to tackling the menace of brucellosis by having a holistic approach into the prevalence of brucellosis in both humans and their livestock in the same household [5, 6] and by extension, identifying factors that may additionally lead to significant socio-economic impact. The evaluation of the cost of brucellosis should take into account the cost of the human disease, and for this purpose an investigation of the prevalence of the different Brucella species in humans is required, as well as establishing the direct and indirect cost resulting from the infections in humans. In addition, enhanced support from the government and the private sector, in accessing insecurity-prone areas (such as Baringo County) could sustain brucellosis control campaigns, as they also need to benefit from these control measures.

## Conclusion

Even though there was a huge socio-economic impact on milk production, infertility, sale value, and dowry, it was the costs of treatment that was significantly impacted on all symptoms associated with brucellosis on this community. A ‘One Health’ approach to tackling the brucellosis menace as a holistic approach is urgently needed to address the prevalence of brucellosis in both humans and their livestock in the same households.

## Methods

### Study area

Baringo County is situated in the Rift Valley Region and shares borders with 8 counties namely, West Pokot to the North West, Turkana to the North, Samburu to the North East, Laikipia to the East, Nakuru to the South, Kericho and Uasin-Gishu Counties to the South West, and Elgeyo-Marakwet to the West. The County is divided into 6 Sub-Counties, namely Baringo South, Mogotio, Eldama Ravine, Baringo Central, Baringo North and Tiaty. It is predominantly inhabited by the Tugen, Pokot and Ilchamus ethnic groups, who are livestock keepers; with minority groups such as Endorois, Nubians, Ogiek, Kikuyu and Turkana. The Tugens mostly practice agro-pastoralism. This mixture of land use allows for complex human-animal interactions usually compounded by the high population density and diversity [15]. It is these complex dynamics that our study was aiming to unravel with respect to brucellosis. Baringo County is classified as arid and semi-arid. Most parts of East Pokot, Baringo Central, Baringo South, Baringo North and Mogotio sub-counties are arid and semi-arid except for Eldama Ravine Sub-County which is a highland zone. The rainfall varies from 1000 mm to 1500 mm in the highlands to 600 mm per annum in the lowlands. The sub-counties due to their varied altitudes receives different levels of rainfall. Eldama Ravine Sub-County receives the highest amount of rainfall. The lowlands sub-counties of Mogotio, East Pokot and Baringo North receive up to 600 mm of rainfall per year. The region is occupied by nomadic communities that place qualifies it as a higher risk region for brucellosis prevalence.

### Study design

**Fig. 1:**
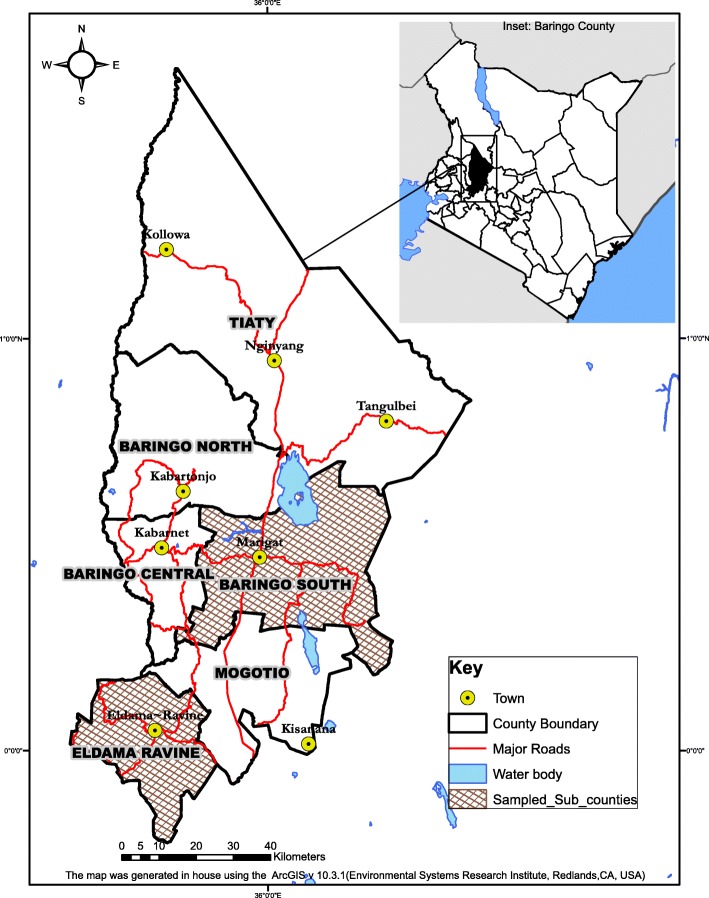
The figure shows the map of the study site within Baringo County. The map was created using ArcGIS® (version 10.31) software by Esri.ArcGIS® and ArcMap™ are the intellectual property of Esri and are used herein under license. Copyright© Esri. All rights reserved. For more information about Esri® software, please visit www.esri.com

The study was carried out in Koibatek (in Eldama Ravine Sub-County) and Marigat (in Baringo South Sub-County) within Baringo County (See Fig. 1). These 2 regions were purposively selected since they were the only agro-pastoral (Koibatek) and arid (Marigat) communities in Baringo County. To realize the objectives of this research, a cross-sectional study applying quantitative approach of data collection was adopted.

#### Study population and sampling procedures

##### Study population

The study population consisted of farmers, herders and their livestock (sheep, goats, cattle and camels) from Koibatek and Marigat sub-counties. Majority of livestock were owned by pastoralists who migrate throughout the dry season looking for pastures in small groups of families or in large groups of villages [15]. In effect, the herds in each grouping can be owned by more than one family, but usually it is a herd per family.

##### Sample size determination

The sample size was determined by Cochran formula [22] which allowed for the calculation of an ideal sample size given a desired level of precision, confidence interval and the estimated proportion of attribute present in the population. Population proportion estimated to the prevalence of brucellosis was 30% in an animal herd, an absolute precision of 5% and at 95% confidence level adopted for this study. Based on these estimates, the final sample size obtained was 640 herds of domestic ruminants comprising of 320 bovines, 154 goats, 106 camels and 60 sheep in 604 households.

##### Sampling procedure

Probability sampling techniques using cluster and simple random methods was used to practically access households that had domestic ruminants. A random sample of 50 villages was selected using a table of random numbers which gave 30 pastoral villages from Marigat and 20 agro-pastoral villages from Koibatek. The team, trained on the data collection instruments and ethical issues, comprised of two enumerators who covered at least one village in a day to administer a minimum of 8 questionnaires at random and these were uploaded in Open Data Kit (ODK) in real-time. Once the team was within the prescribed geocode, the compound to be assessed was identified using the ‘spin bottle method.’ Using a flat surface, the enumerator could spin the bottle until it settled and take the direction facing the mouth of the bottle until he/she reached a household with a domestic ruminant. The first household in that direction was selected and the team entered into the household to administer the questionnaire. The consent was first sought from respondent before proceeding with the questionnaire administration.

Once the respondent in the first household gave consent and agreed to be interviewed by the enumerator, the enumerator, stood at the door of the just completed house and spun the bottle again to pick the direction of the mouth of the next bottle. The enumerator again walked to the next household until all eight eligible households were interviewed. An enumerator that reached the end of the village before completing the numbers required, went back to the center of the village and span the bottle once again. In case the enumerator double-selected the previous household, that household was excluded and the exercise was repeated until another eligible household with domestic ruminants was selected.

#### Methods of data collection

Quantitative data was collected using the Open Data Kit (ODK) software that captured the perceived socio-economic effects on livestock production and reproduction performance. These were pre-tested and customized accordingly prior to actual administration. The data collection exercise was conducted in Tugen, the local language, Kiswahili or in special cases, where the respondent was knowledgeable, in English.

##### Questionnaire interview method

The questionnaire was administered to each respondent by an enumerator for a period of 35 min. During the interview process, focus was on: respondent’s perceived socio-economic impact of brucellosis on livestock production and reproduction performance with a focus on milk, meat and blood, and fat production, infertility, sale value, dowry and cost of treatment relative to suspected brucellosis symptoms (abortions, still births, swollen testes, weak calf or lamb, and swollen joints) (See Supplementary file 1).

### Statistical analysis

Data analysis adopted the use of descriptive and inferential statistics. Descriptive statistics were used to characterize different frequencies. Pearson Chi-square was used to establish the proportionality between the parameters. Logistic regression was further used to establish associations between the symptoms (suspected brucellosis symptoms) and likelihood impacts on milk, meat and blood, and fat production, infertility, sale value, dowry and cost of treatment. All *P*-values ≤0.05 were considered statistically significant.

## Supplementary information

**Additional file 1.** This is the raw data generated from the quantitative questionnaire administered to the respondents.

## Data Availability

The datasets used and/or analyzed during the current study are available from the corresponding author on reasonable request.

## Abbreviations

CI: Confidence interval
FAO: Food and Agricultural Organizations
LMICs: Low- and middle-income countries
ODK: Open data kit
OIE: World Organization for Animal Health
OR: Odd ratio
